# Supplementary material for: Genomic prediction reveals unexplored variation in grain protein and lysine content across a vast winter wheat genebank collection
Source: Front Plant Sci. 2024 Jan 11;14:1270298. doi: 10.3389/fpls.2023.1270298 (PMC10808176; doi:10.3389/fpls.2023.1270298)
Supplement: Supplementary file 1 [file DataSheet_1.pdf]

Supplementary Material

1 Supplementary Figures and Tables

1.1 Supplementary Tables

Table S1 Number of accessions tested in 1 to 7 years for protein content and lysine content.

	Number of years with records						
**Trait**	**1**	**2**	**3**	**4**	**5**	**6**	**7**
**Protein content**	4,422	439	41	19	37	11	1
**Lysine content**	4,423	439	43	21	36	8	1

Table S2 Description of 24 accessions of wheat which were identified by a culling levels selection based on the predicted protein content and adjusted lysine content. Given is the information about the taxonomic variety of *Triticum aestivum* L., the origin (Country, collecting site, altitude [m ASL]) as well as the best linear unbiased estimations (BLUE) and predictions for protein content [%], lysine content [‰] and adjusted lysine content [‰]. Two different levels of selection intensity were applied, given by the z-score. Information about taxonomy and location of origin derive from Oppermann (2023) and the altitude of collecting site derives from Oppermann (2023) and Witcombe (1975).

Accession	Taxonomic description	Collecting site			Protein content		Lysine content		Adjusted lysine content		Selection
	Subtaxon (var.)	Country	Site	Altitude	BLUE	Prediction	BLUE	Prediction	BLUE	Prediction	z-score
TRI 10958	villosum (Alef.) Mansf.	NPL	Gudi Gomba	2,675	21.56	20.76	4.93	4.77	4.54	4.42	0.999
TRI 10956	villosum (Alef.) Mansf.	NPL	Chumisur	2,750	21	20.48	4.94	4.8	4.58	4.46	0.999
TRI 10962	lutescens (Alef.) Mansf.	NPL	Hatia	1,925	20.48	20.41	4.88	4.73	4.56	4.41	0.999
TRI 10471		AFG		2,150	21.77	20.26	4.88	4.7	4.58	4.49	0.999
TRI 10951	heraticum (Kobelev) Mansf.	NPL	Ritak	2,975	20.92	20.07	4.82	4.67	4.49	4.39	0.999
TRI 10957	lutescens (Alef.) Mansf.	NPL	Chumisur	2,750	21.28	20.72	4.72	4.72	4.34	4.37	0.99
TRI 10881	villosum (Alef.) Mansf.	NPL	Tenchong	1,675		20.64		4.7		4.36	0.99
TRI 10927	villosum (Alef.) Mansf.	NPL	Junbesi	2,675		20.63		4.72		4.37	0.99
TRI 11153	pyrothrix (Alef.) Mansf.	NPL	Pangu	2,760		20.61		4.69		4.34	0.99
TRI 11155	villosum (Alef.) Mansf.	NPL	Ghat	2,485		20.51		4.68		4.35	0.99
TRI 10935	villosum (Alef.) Mansf.	NPL	Kosrobas	2,330		20.39		4.68		4.36	0.99
TRI 10911	villosum (Alef.) Mansf.	NPL	Kharikola	1,890		20.33		4.68		4.36	0.99
TRI 11158	villosum (Alef.) Mansf.	NPL	Peingorra	2,530		20.33		4.67		4.36	0.99
TRI 11143	hostianum (Clemente) Mansf.	NPL	Nurbugaon	2,040		20.2		4.64		4.34	0.99
TRI 10952		NPL	Ritak	2,975		20.15		4.64		4.35	0.99
TRI 11160	villosum (Alef.) Mansf.	NPL	Chepua	2,125		20.15		4.65		4.36	0.99
TRI 10909	villosum (Alef.) Mansf.	NPL	Poyam	2,745		20.03		4.64		4.36	0.99
TRI 10915	lutinflatum (Flaksb.) Mansf.	NPL	Chaunrikharka	2,665	19.83	19.7	4.75	4.64	4.48	4.38	0.99
TRI 10834	ferrugineum (Alef.) Mansf.	GRC		850	19.62	19.06	4.65	4.49	4.5	4.38	0.99
TRI 10923	rufinflatum (Flaksb.) Mansf.	NPL	Gonba	2,805	18.16	19.03	4.46	4.52	4.35	4.33	0.99
TRI 10049	ferrugineumcompactoides (Kobelev) Mansf.	GBR			19.63	18.84	4.48	4.55	4.23	4.39	0.99
TRI 10493	submeridionaleinflatum (Palmova) Mansf.	AFG	Faizabad	1,000	19.17	18.84	4.92	4.6	4.79	4.45	0.99
TRI 196	aestivum	YUG			19.63	18.69	4.6	4.52	4.4	4.39	0.99
TRI 14318	erinaceum (Desv.) Mansf.					18.63		4.52		4.39	0.99

1.2 Supplementary Figures

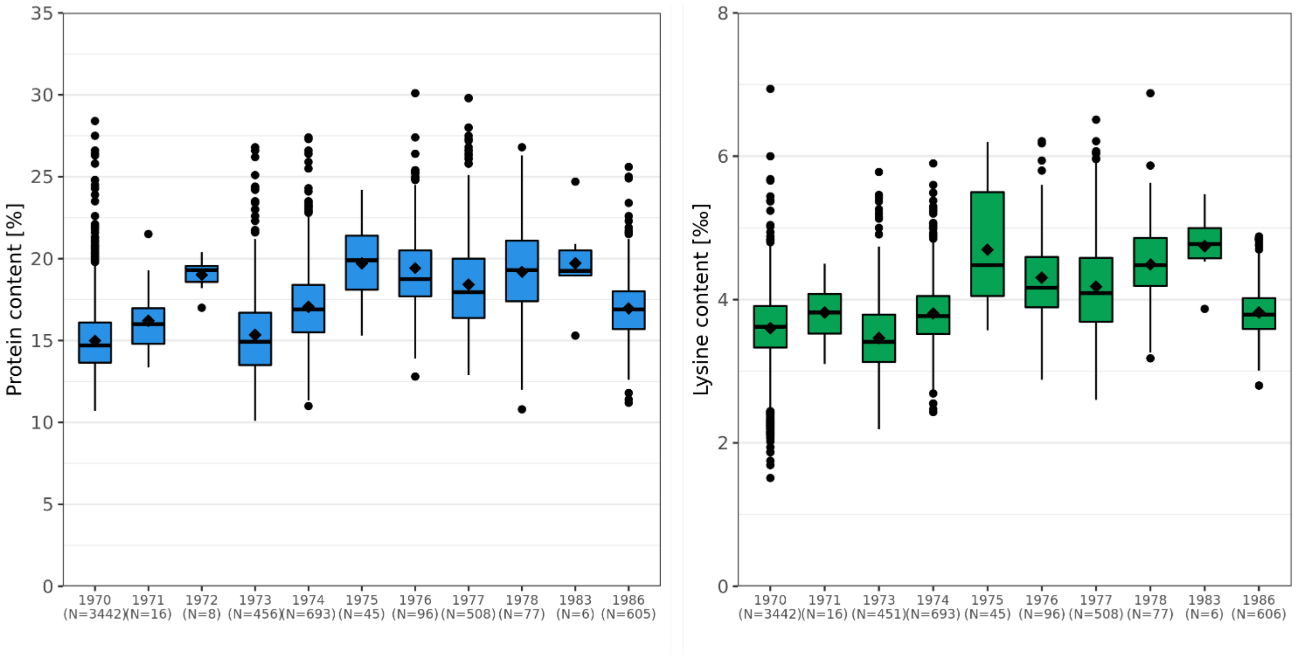

Fig. S1 Distribution of protein content and lysine content separately per year of cultivation. Shown are the raw phenotypic values for the period 1970-1986. The number of different measurements (N) is displayed on the x-axis. Boxes enclose 50% of the central data, including median (horizontal black bold line) and mean (black diamonds), while whiskers are ± 1.5 × interquartile range and dots represent extreme values.

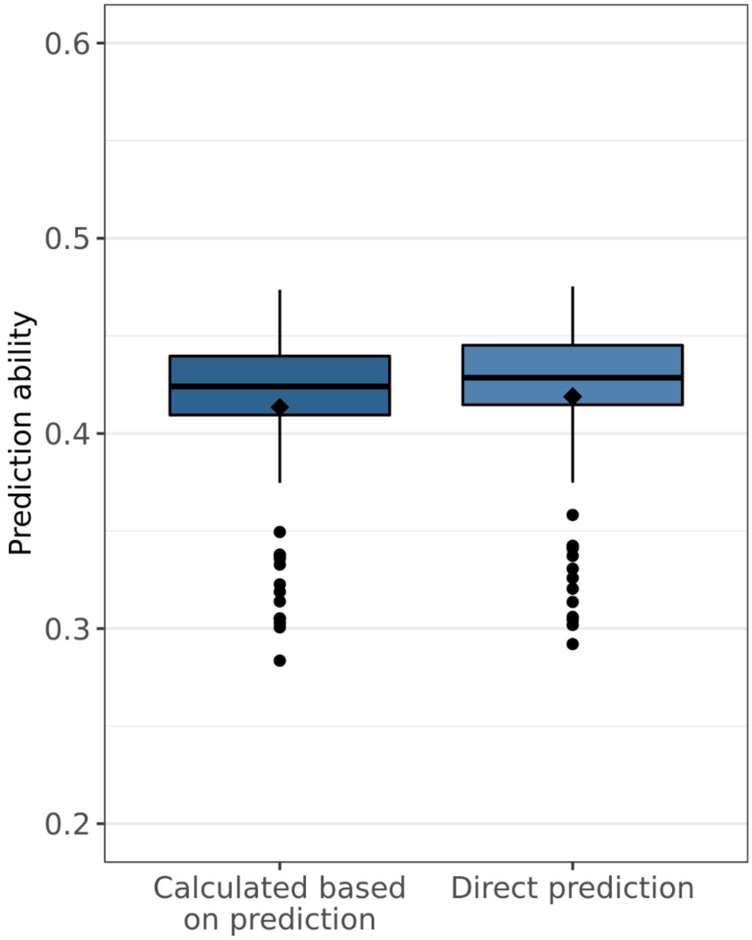

Fig. S2 Prediction abilities for two approaches to predict the derived trait adjusted lysine content. Prediction abilities were estimated using 100 runs of five-fold cross-validations. In the first approach, adjusted lysine content is calculated based on the predictions of lysine content, protein content, and thousand grain weight. In the second approach, adjusted lysine content is predicted based on the adjusted lysine content calculated from the best linear unbiased estimates. Boxes enclose 50% of the central data, including median (horizontal black bold line) and mean (black diamonds), while whiskers are ± 1.5 × interquartile range and dots represent extreme values.

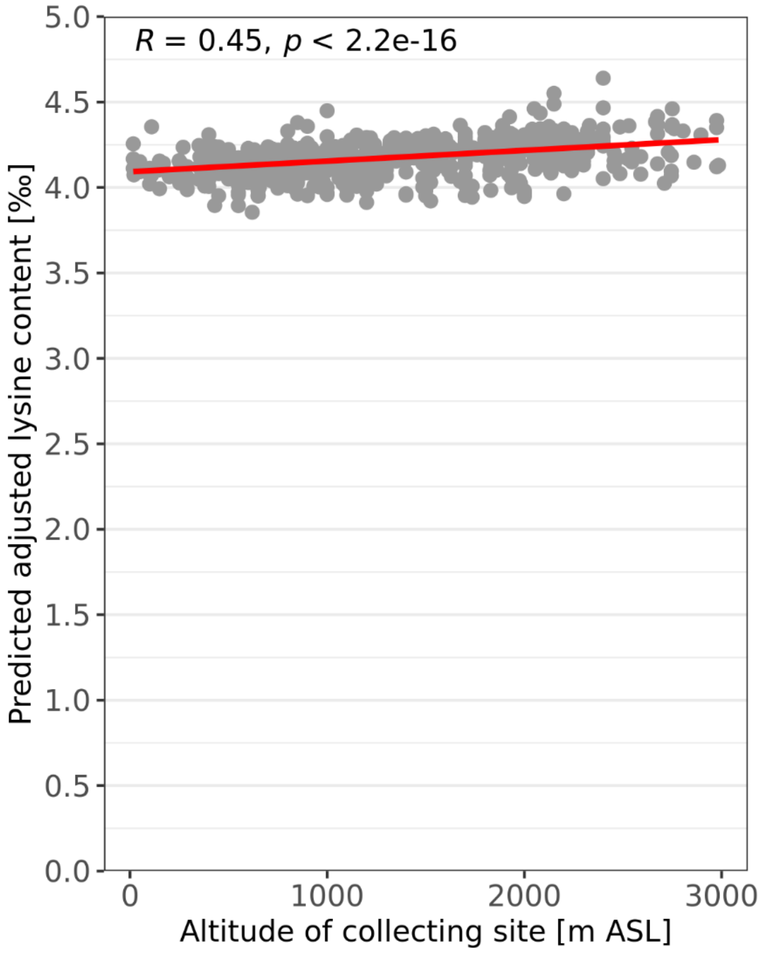

Fig. S3 Predicted adjusted lysine content in per mille in association with the altitude of the collecting site in meter above sea level. Shown are the values of 927 accessions.
